# HLA-C -35kb Expression SNP Is Associated with Differential Control of β-HPV Infection in Squamous Cell Carcinoma Cases and Controls

**DOI:** 10.1371/journal.pone.0103710

**Published:** 2014-08-01

**Authors:** Karin A. Vineretsky, Margaret R. Karagas, Jacquelyn K. Kuriger-Laber, Tim Waterboer, Michael Pawlita, Heather H. Nelson

**Affiliations:** 1 Department of Environmental Health Sciences, School of Public Health, University of Minnesota, Minneapolis, Minnesota, United States of America; 2 Department of Community and Family Medicine, Norris Cotton Cancer Center, Geisel School of Medicine at Dartmouth, Hanover, New Hampshire, United States of America; 3 German Cancer Research Center (DKFZ), Heidelberg, Germany; 4 Division of Epidemiology and Community Health, School of Public Health, University of Minnesota, Minneapolis, Minnesota, United States of America; 5 Masonic Cancer Center, University of Minnesota, Minneapolis, Minnesota, United States of America; University of Missouri Kansas City School of Medicine, United States of America

## Abstract

A single nucleotide polymorphism (SNP) 35 kb upstream of the HLA-C gene is associated with HLA-C expression, and the high expressing genotype (CC) has been associated with HIV-I control. HLA-C is unique among the classical MHC class I molecules for its role in the control of viral infections and recognition of abnormal or missing self. This immunosurveillance is central to the pathogenesis of non-melanoma skin cancer (NMSC), and of squamous cell carcinoma (SCC) in particular. While sun exposure is a major risk factor for these cancers, cutaneous infections with genus β-HPV have been implicated in the development of SCC. We hypothesized that the high expression HLA-C genotype is associated with β-HPV infections. Therefore, we investigated the association between β-HPV serology and the −35 kb SNP (rs9264942) in a population-based case-control study of 510 SCC cases and 608 controls. Among controls, the high expression −35 kb SNP genotype (CC) reduced the likelihood of positive serology for multiple (≥2) β-HPV infections (OR = 0.49, 95% CI: 0.25–0.97), and β-HPV species 2 infection (OR = 0.43, 95% CI: 0.23–0.79). However, no association with β-HPV status was observed among SCC cases. Our findings suggest that underlying immunogenotype plays an important role in differential control of β-HPV in SCC cases and controls.

## Introduction

Non-melanoma skin cancers (NMSC) represent the most common malignancies in the world with increasing incidence rates [1], [2]. NMSC include basal cell carcinoma (BCC) and squamous cell carcinoma (SCC), representing about 80% and 20% of disease burden, respectively [3]. While ultra violet radiation (UVR) is a major risk factor for NMSC, exposure to cutaneous genus beta human papilloma viruses (β-HPV) has been associated with the pathogenesis of SCC [4]. Central to the pathogenesis of NMSC is the ability of the immune system to identify and eliminate abnormal cells, including those harboring HPV infection.

The connection between immunity and NMSC is well described in individuals undergoing immunosuppressive therapies [2], [5]. The immunosurveillance of healthy skin is achieved by an extensive network of immune cells [6], with evidence suggesting that resident rather than recruited T cells play a major role in local skin immunity [7], [8]. The keratinocytes also play a key role in innate skin defense by producing immunomodulating chemokines and cytokines, and expressing a variety of pattern recognition receptors, including toll-like receptors, as well as HLA molecules of the major histocompatibility complex (MHC) class I [9], [10], [11]. Furthermore, the unconventional innate-like γδ T cells and natural killer (NK) cells have been proposed to reside in healthy skin [6]. Like the NK cells, γδ T cells exhibit rapid response to antigens, stress-associated factors such as heat shock proteins, and express activating natural killer group 2 member D (NKG2D) receptors which respond to tumor or virally induced MHC I chain-related stress proteins MICA and MICB [12],[13]. Deregulation of the innate and T cell-mediated immune responses are implicated in a number of autoimmune disorders and skin pathologies [6], [14], [15]. This includes the rare genetic disorder Epidermodysplasia Verruciformis (EV) that is associated with extreme susceptibility to persistent β-HPV infections and increased SCC risk [4], [16].

Cutaneous HPV include alpha, beta, gamma, mu, and nu genera, which are further grouped into species, type, and subtype, according to the extent of sequence identity [18], [19]. Genus beta HPV include species 1–5, and are common in the general population as latent or unapparent infections, and immunocompetent individuals may carry multiple species and types of β-HPV [16], [17], [18]. Furthermore, seropositivity to multiple β-HPV types is a risk trait for SCC [20]. Abnormal cell-mediated immunity has been implicated in the pathogenesis of EV [16], [21], [22], [23] and common genetic variation in the EV-related gene *TMC8* is associated with seropositivity to multiple β-HPV types among healthy individuals [24]. Additional immunogenetic traits likely influence the control of these cutaneous infections.

HLA-C is unique among the classical MHC class I molecules for its role in the control of viral infections and recognition of abnormal or missing self, by presenting antigens to cytotoxic T lymphocytes (CTL) and providing an inhibitory signal to NK cells [14], [25]. Another distinct feature of HLA-C molecules is the maintenance of low surface expression levels via transcriptional, translational, and post-translational regulation [25]. The fine tuned control of HLA-C expression is central for an effective immune response. Recent studies have shown that rapid turnover of the HLA-C heavy chain mRNA is regulated by a micro RNA binding site, miRNA-148a [25], [26]. A deletion/insertion polymorphism at position 263 downstream of the HLA-C stop codon affects the binding of this miRNA and is associated with differential surface expression of HLA-C [25]. A SNP (rs9264942) in linkage disequilibrium (LD) with position 263 has been identified as key in HIV control with the (CC) genotype associated with higher HLA-C expression, lower HIV viral load set point and protective phenotype [25], [26], [27]. We hypothesized that similar immune interactions may be applicable in the context of β-HPV infections, with differential β-HPV control and susceptibility that is dependent on an individual’s immune response tagged by the −35 kb HLA-C expression SNP. Therefore, we investigated the association of −35 kb HLA-C expression SNP immunogenotype with β-HPV serology in SCC cases and controls.

## Materials and Methods

### Ethics statement

Study protocols and materials for recruitment of cases and controls were approved by the Committee for the Protection of Human Subjects at Dartmouth College. Informed consent was obtained from all study subjects.

### Study population

Newly diagnosed cases of histologically confirmed SCC in New Hampshire were identified using an incident survey as previously described [28], [29]. Controls were randomly selected from the resident population and were frequency matched to cases on age and gender. Controls <65 years of age were derived from the New Hampshire Department of Transportation, and those >65 years of age derived from Medicare enrollment records. The participants were administered an interview including demographic factors, pigmentation characteristics, sun exposure and sensitivity, and other factors as previously described [30], [31]. Overall, 1,084 SCC cases were identified in the incident survey, of these 1036 (96%) were contacted and eligibility confirmed, and 833 (80%) were interviewed. Of the 1,527 potential controls, 1,462 (96%) were contacted and confirmed as eligible and 1,066 (73%) of those were interviewed [20]. Of these enrolled participants a plasma sample was available for 80% of the cases and 76% of the controls, and DNA was available for 77% of the cases and 71% of the controls. No appreciable differences were noted comparing those who did and did not have molecular marker data (data not shown).

### Sample collection and β-HPV serology

From all participants a venous blood sample of 20–30 mL was collected in heparinized tubes. Blood was separated by centrifugation at 2,500 *g* for 20 min at 4°C. Plasma, red blood cells and the buffy coat were stored separately at −80°C until analysis. Each specimen was labeled with a type code (plasma, red blood cells or buffy coat) and a unique identifier so that the staff was masked to the disease status associated with all samples. Frozen plasma samples were previously analyzed for antibodies to the major capsid protein L1 of genus β human papillomavirus types 5, 8, 9, 15, 17, 20, 23, 24, 36, 38, 49, 75, 76, 92, 96, and 107 [4]. The antibody detection method is based on a glutathione *S*-transferase capture enzyme-linked immunosorbent assay (ELISA), in combination with fluorescent bead technology [32], [33], [34].

### HLA-C −35 kb SNP genotyping

DNA was extracted from buffy coat using Qiagen genomic DNA extraction kits. We collected data on (T>C) coding SNP located in the 5′ region of the HLA-C gene, 35 kb away from the transcription initiation site (rs9264942), which is a marker of level of HLA-C messenger RNA transcript and cell surface expression. Genotyping of the −35 kb SNP polymorphism was performed using the Taqman allelic discrimination technique (Applied Biosystems, Foster City, CA).

### Statistical analysis

We examined the association of the −35 kb SNP genotype with seropositivity to β-HPV among SCC cases and controls. This included an analysis of multiple β-HPV (≥2) and β species: β1 (5, 8, 20, 24, 36), β2 (9, 15, 17, 23, 38, 107), and β3 (49, 75, 76). In addition we tested the association of the −35 kb SNP genotype with case status, including an analysis stratified by β-HPV species. For all models odds ratios (OR) and 95% confidence intervals (95% CI) were calculated using unconditional logistic regression, adjusted for age at diagnosis, sex, and skin type. The measure of skin type was defined as the reaction to 1 hour of sun exposure the first time in the summer. For greater statistical power the four level response variable was collapsed to two levels; those who responded that they had a severe sunburn with blistering, or painful sunburn for a few days followed by peeling were assigned in the Burn category, and those that responded that they tanned without any sunburn, or had a mild burn followed by tanning, were assigned Tan category [4], [20], [35]. All statistical analyses were performed using R v2.13.1.

## Results

Genotype data for the −35 SNP were available for 1118 white participants, 608 controls, and 510 SCC cases. Allele frequencies were in Hardy-Weinberg equilibrium. The prevalence of the C allele was 39% in controls and 38% in the SCC group, consistent with the published prevalence in white populations [36], [37]. The demographic and exposure characteristics of the participants are presented in Table 1. We examined the association of the −35 kb SNP genotype with β-HPV status among SCC cases and controls, comparing those with no antibody positivity for β-HPV to those with any positivity, and individuals with multiple β-HPV types (Table 2). In controls, the high expression −35 kb SNP genotype (CC) reduced the likelihood of positive serology for multiple (≥2) β-HPV infections (OR = 0.49, 95% CI: 0.25–0.97). However, no significant association was found in the SCC group (OR = 0.86, 95% CI: 0.47–1.60) (Table 2).

**Table 1:** Demographic characteristics of the study participants.

	Total	Controls	SCC
**N (%)**	1118	608 (54)	510 (46)
**Age**			
**Mean (SD)**	63 (10)	62 (10)	64 (9)
**Median (Range)**	66 (28–74)	65 (28–74)	67 (31–74)
**Sex**			
**Male (%)**	717 (64)	379 (62)	338 (66)
**Female (%)**	401 (36)	229 (38)	172 (34)
**Skin Type**			
**Burn (%)**	415 (37)	188 (31)	227 (45)
**Tan (%)**	699 (63)	419 (69)	280 (55)
**Missing (%)**	4 (0.4)	1 (0.1)	3 (0.5)

**Table 2:** High expression genotype (CC) and β-HPV positive serology among controls and SCC cases.

Controls										
	0 β-HPV		≥1 β-HPV				≥2 β-HPV			
	N = 327	(%)	n = 281	(%)	OR	(95%CI)	N = 159	(%)	OR	(95%CI)
***−35 SNP (TT)***	125	(38)	101	(36)	Ref.		63	(40)	Ref.	
***−35 SNP (CT)***	149	(46)	146	(52)	1.20	(0.85–1.71)	83	(52)	1.10	(0.73–1.64)
***−35 SNP (CC)***	53	(16)	34	(12)	0.80	(0.48–1.33)	13	(8)	0.49	(0.25–0.97)
**SCC**										
	**0 β-HPV**		**≥1 β-HPV**				**≥2 β-HPV**			
	**N = 233**	**(%)**	**N = 277**	**(%)**	**OR**	**(95%CI)**	**N = 178**	**(%)**	**OR**	**(95%CI)**
***−35 SNP (TT)***	96	(41)	106	(38)	Ref.		68	(38)	Ref.	
***−35 SNP (CT)***	100	(43)	132	(48)	1.17	(0.80–1.71)	87	(49)	1.21	(0.79–1.85)
***−35 SNP (CC)***	37	(16)	39	(14)	0.93	(0.54–1.58)	23	(13)	0.86	(0.47–1.60)

Next, we examined the association of the −35 kb SNP genotype with β-HPV positive serology stratified by β-HPV species (1, 2 and 3), among controls and SCC cases (Table 3). In controls, the high expression −35 kb SNP genotype (CC) was significantly associated with decreased risk of β-HPV species 2 seropositivity (OR = 0.43, 95% CI: 0.23–0.79). No association of the −35 kb SNP genotype with species specific positivity was found in SCC (Table 3).

**Table 3:** Among controls the high expression genotype (CC) decreases the likelihood of Beta HPV species 2 seropositivity.

β-HPV species	Controls							SCC					
β1													
	0		≥1				0			≥1			
	n = 470	(%)	n = 138	(%)	OR	(95%CI)	n = 342		(%)	n = 168	(%)	OR	(95%CI)
***−35 SNP (TT)***	180	(38)	46	(33)	Ref.		139		(41)	63	(38)	Ref.	
***−35 SNP (CT)***	220	(47)	75	(54)	1.35	(0.89–2.06)	147		(43)	85	(51)	1.25	(0.84–1.87)
***−35 SNP (CC)***	70	(15)	17	(12)	1.03	(0.55–1.92)	56		(16)	20	(12)	0.80	(0.44–1.44)
**β2**													
	**0**		**≥1**				**0**			**≥1**			
	**n = 402**	**(%)**	**n = 206**	**(%)**	**OR**	**(95%CI)**	**n = 309**		**(%)**	**n = 201**	**(%)**	**OR**	**(95%CI)**
***−35 SNP (TT)***	149	(37)	77	(37)	Ref.		120		(39)	82	(41)	Ref.	
***−35 SNP (CT)***	182	(45)	113	(55)	1.19	(0.83–1.70)	145		(47)	87	(43)	0.86	(0.58–1.27)
***−35 SNP (CC)***	71	(18)	16	(8)	0.43	(0.23–0.79)	44		(14)	32	(16)	1.04	(0.60–1.78)
**β3**													
	**0**		**≥1**				**0**			**≥1**			
	**n = 502**	**(%)**	**n = 106**	**(%)**	**OR**	**(95%CI)**	**n = 415**		**(%)**	**n = 95**	**(%)**	**OR**	**(95%CI)**
***−35 SNP (TT)***	188	(38)	38	(36)	Ref.		162		(39)	40	(42)	Ref.	
***−35 SNP (CT)***	239	(48)	56	(53)	1.16	(0.74–1.83)	189		(46)	43	(45)	0.92	(0.57–1.48)
***−35 SNP (CC)***	75	(15)	12	(11)	0.83	(0.41–1.68)	64		(15)	12	(13)	0.78	(0.38–1.58)

Finally, we investigated the association of the −35 kb SNP genotype with SCC case risk. No significant differences in the prevalence of the C allele were observed between SCC cases and controls (Table 4). Stratification by β-HPV status did not reveal significant differences in the prevalence of −35 kb SNP genotypes between SCC cases and controls among those β-HPV negative (Table 5). However, an elevated risk of SCC with (CC) genotype was observed in the multiple β-HPV and β-HPV species 2 strata, albeit with wide confidence intervals (Table 5).

**Table 4:** Association of −35 kb SNP with case status.

	Controls		SCC			
	N = 608	(%)	N = 510	(%)	OR	(95%CI)
***−35 SNP (TT)***	226	(37)	202	(40)	Ref.	
***−35 SNP (CT)***	295	(49)	232	(45)	0.87	(0.67–1.13)
***−35 SNP (CC)***	87	(14)	76	(15)	1.02	(0.70–1.47)

**Table 5:** Association of −35 kb SNP with case status, stratified by β-HPV.

	Controls		SCC			
Number of β-HPV	N	(%)	N	(%)	OR	(95%CI)
**0**						
***−35 SNP (TT)***	125	(38)	96	(41)	Ref.	
***−35 SNP (CT)***	149	(46)	100	(43)	0.88	(0.61–1.28)
***−35 SNP (CC)***	53	(16)	37	(16)	1.00	(0.60–1.66)
**≥1**						
***−35 SNP (TT)***	101	(36)	106	(38)	Ref.	
***−35 SNP (CT)***	146	(52)	132	(48)	0.83	(0.57–1.21)
***−35 SNP (CC)***	34	(12)	39	(14)	1.04	(0.60–1.81)
**≥2**						
***−35 SNP (TT)***	63	(40)	68	(38)	Ref.	
***−35 SNP (CT)***	83	(52)	87	(49)	0.96	(0.60–1.54)
***−35 SNP (CC)***	13	(8)	23	(13)	1.87	(0.84–4.15)
**≥2 β-HPV Species 2**						
***−35 SNP (TT)***	46	(42)	42	(39)	Ref.	
***−35 SNP (CT)***	54	(50)	51	(47)	0.98	(0.54–1.77)
***−35 SNP (CC)***	9	(9)	15	(14)	2.04	(0.76–5.47)

## Discussion

We investigated the association of an HLA-C expression genotype with presence of β-HPV seropositivity in SCC cases and controls. Among controls the high expression (CC) genotype was associated with reduced prevalence of multiple β-HPV and species 2 β-HPV serologic response, compared to controls with negative β-HPV serology. However, no significant associations with β-HPV status were observed among SCC cases. Our findings indicate that the −35 kb SNP is implicated in β-HPV control in healthy individuals, but not in SCC patients. This suggests a deregulation of HLA-C mediated immunity in individuals with SCC, that potentially relates to the development of skin tumors.

Previous studies of the HLA-C −35 kb expression SNP have been focused on HIV infection control. While β-HPV and HIV infections are different in their mode of action and target cell type, the initial immune response in early viral control and maintenance of chronic infection are important for both. In the context of HIV, the protective effect of the high expressing (CC) genotype has been attributed to better antigen presentation to CTL, especially in early HIV control, and effective education and licensing of the maturing NK cells [27]. We cannot specifically attribute the inverse association with the (CC) genotype observed in our study to either the CTL or NK cell mediated response. However, given the complexity of interactions in the setting of multiple β-HPV seropositivity, an efficient antigen presentation in the skin, as well as target recognition and destruction by the NK cells or the unconventional innate-like γδ T cells, are likely important for local infection control. In addition, HLA-C restricted CTL responses have been described in chronic infections such as Epstein–Barr virus, cytomegalovirus, and HIV as reviewed in Blais et. al. [14], and similar interactions may be relevant for an efficient control of β-HPV infection.

Given its dual functionality in interactions with innate and adaptive immunity, HLA-C plays a key role in immunosurveillance of both virally infected cells and recognition and elimination of tumor transformed cells. Tumor immunosurveillance is highlighted in the immunosupressed OTR and EV patients with deregulated innate and cell mediated immune responses [2], [5], [15]. These patients are at high risk of SCC development and are frequently infected with genus β-HPV, and β-HPV species 1 in particular [2], [5], [15], [23], [38], [39], [40]. Many β-HPV types, including β-HPV species 2, are prevalent in immunocompetent individuals and constitute a part of the normal skin flora, have been linked to an increased SCC risk [4], [17], [41], [42], [43]. Previous investigation of our study population by Farzan et. al. demonstrated an enhanced risk of SCC with enrichment for β-HPV species 2 types 9, 15, 17, 23, 38, and 107 [20]. In addition multiplicity of β-HPV was also associated with an increased SCC risk [20]. Our results suggest that HLA-C may contribute to the control of multiple β-HPV in healthy individuals but not in those who develop SCC.

While the mechanism of SCC development and its relationship to β-HPV positivity is yet to be determined, our results show a lack of association between HLA-C expression genotype and β-HPV among SCC cases, suggesting a potential deregulation of HLA-C mediated immunosurveillance. This is further corroborated by our β-HPV stratified case-control analysis, where SCC was positively associated with genotype only among those with multiple or β-HPV species 2 positive serology, an association driven by differences in genotype distribution among controls and not the cases. Thus, the underlying immunogenotype, which is implicated in control of β-HPV, may be a potential susceptibility factor for SCC, and individuals with this underlying deregulated immunity may be predisposed to the development of tumors.

Interestingly, the HLA-C locus has been reported as a major determinant of susceptibility to psoriasis, an inflammatory skin disorder with a strong genetic component, and the low expressing T allele of the −35 kb SNP is associated with an increased risk of the disease [44], [45]. Moreover, genetic variants that are protective in HIV controllers are over-represented in psoriasis patients, suggesting an evolutionary trade-off between autoimmunity and anti-viral protection [45]. Our observations of enhanced β-HPV control among those with the (CC) genotype are consistent with both HIV protection and those reported with psoriasis. Among controls, those with (CC) genotype were less likely to have multiple positive serology and species 2 β-HPV serology, indicating functional immunity and efficient viral control. This suggests that “normal” HLA-C function and associated immune interactions are deregulated or potentially absent in SCC, and therefore do not modify the risk of β-HPV, in contrast to observations in SCC-free controls. In simple terms, compared to the “hyperactivated” immunophenotype in psoriasis and “normal” state in healthy controls, the SCC group may represent an immunophenotype with a suppressed immune state where antiviral and antitumor responses are deregulated.

Previous investigations of β-HPV control have shown a link between common genetic variation and positive β-HPV serology [24], [46]. Patel et. al. reported that a common variant in TMC8 (rs7208422) was associated with both an increased prevalence of β-HPV infections and increased risk of SCC [24]. TMC8, first recognized for its association with the rare disorder Epidermodysplasia Verricuformis, is expressed at high levels in both keratinocytes and T-lymphocytes [47], [48]. In addition, a recent genome-wide association study described a variant within the MHC II region (rs9357152) that was associated with positive serology for β-HPV type 8 [46]. The Chen et. al. study did not find an association between the HLA-C variant described here and β-HPV serology. This lack of association may be due to the limited number of HPV types tested in their study design, and the inability to look at the multiple-HPV endpoint. Together, these studies support a genetic basis for immune control of β-HPV, and the involvement of both the MHC-I and MHC-II.

In this study we only measured a single genetic marker, the −35 kb SNP. The MHC-I region and the polymorphic HLA-C loci contain extensive LD. Moreover, −35 kb SNP has been shown to be in partial LD with the HLA complex P5 variant, which, in turn, is in strong LD with the HLA-B*5701 allele that is strongly associated with viral control of HIV [36]. Thus, −35 kb SNP may only serve as a proxy of the complexity involved in HLA-C regulation. It will be important for future investigations to study immunogenetics in greater depth including HLA-C alleles, NK cell receptors, as well as regulatory cytokine markers. HLA-C is important in interactions with both the innate and cell-mediated components of the immune response, in early and chronic viral control, as well as recognition and elimination of tumors. Our results indicate a differential control of β-HPV in SCC cases and controls, potentially attributable to the underlying immunogenotype.
